# Pediatric central venous access devices: practice, performance, and costs

**DOI:** 10.1038/s41390-022-01977-1

**Published:** 2022-02-08

**Authors:** Amanda J. Ullman, Victoria Gibson, Mari D. Takashima, Tricia M. Kleidon, Jessica Schults, Masnoon Saiyed, Paula Cattanach, Rebecca Paterson, Marie Cooke, Claire M. Rickard, Joshua Byrnes, Vineet Chopra

**Affiliations:** 1 School of Nursing, Midwifery and Social Work, University of Queensland, Brisbane, QLD Australia; 2 Queensland Children’s Hospital, Brisbane, QLD Australia; 3 Menzies Health Institute Queensland and School of Nursing and Midwifery, Griffith University, Brisbane, QLD Australia; 4 Centre for Applied Health Economics, Griffith University, Brisbane, QLD Australia; 5 Department of Medicine, University of Colorado at Denver, Anschutz Medical Campus, Denver, CO USA

## Abstract

**Background:**

Healthcare delivery is reliant on a functional central venous access device (CVAD), but the knowledge surrounding the burden of pediatric CVAD-associated harm is limited.

**Methods:**

A prospective cohort study at a tertiary-referral pediatric hospital in Australia. Children <18 years undergoing insertion of a CVAD were screened from the operating theatre and intensive care unit records, then assessed bi-weekly for up to 3 months. Outcomes were CVAD failure and complications, and associated healthcare costs (cost of complications).

**Results:**

163 patients with 200 CVADs were recruited and followed for 6993 catheter days, with peripherally inserted central catheters most common (*n* = 119; 60%). CVAD failure occurred in 20% of devices (*n* = 30; 95% CI: 15–26), at an incidence rate (IR) of 5.72 per 1000 catheter days (95% CI: 4.09–7.78). CVAD complications were evident in 43% of all CVADs (*n* = 86; 95% CI: 36–50), at a rate of 12.29 per 1000 catheter days (95% CI: 9.84–15.16). CVAD failure costs were A$826 per episode, and A$165,372 per 1000 CVADs. Comparisons between current and recommended practice revealed inconsistent use of ultrasound guidance for insertion, sub-optimal tip-positioning, and appropriate device selection.

**Conclusions:**

CVAD complications and failures represent substantial burdens to children and healthcare. Future efforts need to focus on the inconsistent use of best practices.

**Impact:**

- Current surveillance of central venous access device (CVAD) performance is likely under-estimating actual burden on pediatric patients and the healthcare system.
- CVAD failure due to complication was evident in 20% of CVADs. Costs associated with CVAD complications average at $2327 (AUD, 2020) per episode.
- Further investment in key diverse practice areas, including new CVAD types, CVAD pathology-based occlusion and dislodgment strategies, the appropriate use of device types, and tip-positioning technologies, will likely lead to extensive benefit.

## Introduction

The insertion of a central venous access device (CVAD) signals the commencement, or re-commencement, of life-changing treatment for children and their families. Often a child’s first significant healthcare procedure, CVADs are a tool of the trade for most pediatric health disciplines—used for treatments varying from the administration of antibiotics for chronic osteomyelitis, to lifelong parenteral nutrition for gut enteropathies. But a child’s healthcare experience is often disrupted by complications caused by how healthcare systems and clinicians select, insert, manage and remove CVADs.^1,2^

CVAD performance can be a measure of hospital performance. However, conventionally only single outcomes or populations are benchmarked—most commonly infections or thromboses, in cancer or intensive care.^3–5^ Other forms of CVAD complications, such as catheter breakage, dislodgement, and occlusion, are rarely collected or compared. This potentially underestimates the true phenomenon of CVAD-associated harm, and the associated burden of CVAD-associated harm for children, their families, and healthcare. It also impairs the ability for clinicians and researchers to effectively benchmark or target impactful and sustainable improvements. Our knowledge surrounding the burden of pediatric CVAD-associated harm is incomplete.^6^

Given the lack of these standardized metrics, the full economic costs of CVAD-associated harm in pediatrics are also unclear. Estimates of attributable costs, including length of hospital stay and catheter-associated bloodstream infections (CABSI) are significant, and have been described for children with cancer (additional 21.2 hospital days [95% confidence interval CI: 10.4–32.0]; $69,332 [2012 USD; 95% CI: 35,144–103,521]),^4^ in the intensive care (additional 19 hospital days [95% CI: 14.3–23.8]; $55,646 [2011 USD; 95% CI: 38,785–72,507]),^7^ and in general pediatrics (additional 21 hospital days [95% CI: 7.3–34.8]; €13,727 [2017 Euro; 95% CI: 5758–21,695]).^8^ But the economic costs of other forms of CVAD harm, that also result in additional procedures, hospital admissions, and treatment disruption are relatively unknown but likely substantial. For example, we have previously conservatively costed repeated complications in one child to have additional healthcare-costs of more than $10,000 (2016 AUD).^1^

The current high rate of complications and cost may stem from non-adherence to best practice guidelines. However, children requiring CVADs are diverse and it is difficult to ensure guidelines are pertinent across all pediatric cohorts. But there are recommendations for care that are supported by high-quality evidence, that should be routinely implemented. For example, ultrasound guidance for CVAD insertion;^9–11^ CVAD tip placement in the cavo-atrial junction;^12^ not using CVADs for a short duration, non-vesicant infusates;^13^ and avoiding totally implanted devices for neonates and infants.^13^ Examining whether these practices have been universally implemented within healthcare services serves two complementary purposes: identifying the need for knowledge translation, and identifying situations where non-routine practice is appropriate, and innovation is required.

To solve a complex problem, we first need to unravel its layers. In this study, we aimed to describe CVAD insertion practices, performance, and healthcare-costs across a large pediatric health service. We also aimed to identify risk for increased pediatric CVAD-associated harm, and explore gaps between current practice and best practice. This will provide a comprehensive explanation of the contemporary practice, performance, and value, towards prioritized, tangible improvements.

## Methods

### Study design

A prospective cohort study was undertaken at a tertiary-referral pediatric hospital in Australia, between September 2018 and March 2020. Data encompassing CVAD insertion and management procedures were prospectively collected, and participants were followed for up to three months to report CVAD performance (including complications and removals) and associated costs. Ethical approvals were obtained from the Children’s Health Queensland and Griffith University Human Research Ethics Committee (HREC/18/QRCH/19; 2018/096). The study is reported in accordance with the Strengthening The Reporting of OBservational studies in Epidemiology (STROBE) guidelines.^14^

### Setting

The study included all clinical areas within the Queensland Children’s Hospital (QCH), Australia. QCH is Queensland’s tertiary-referral pediatric facility, with 359 beds it provides care to patients from maternity hospital discharge to 18 years of age, across all major disciplines (including specialized cardiac services). The QCH does not admit neonates for immediate post-birth management (e.g., prematurity, birth trauma).

### Participants and sample size

All children less than 18 years, undergoing insertion of a CVAD (including peripherally inserted central catheters [PICCs], non-tunneled CVADs, tunneled (with or without a Dacron cuff) CVADs, hemodialysis catheters [HDs], totally implanted venous devices [a.k.a. ports/TIVD]) at the QCH in operating theatres and intensive care within the study period were eligible for inclusion. Children having sub-specialty devices inserted, such as intrathoracic lines, ECMO cannula, and direct hemodialysis methods (i.e., fistulas), were not included.

Due to local resources and to ensure quality follow-up (minimizing missing data), a maximum of only 10 participants could be followed at a time. Whenever a patient finished follow-up (at study end), a new patient was consecutively commenced. To ensure the sample consecutively recruited was representative of the pediatric CVAD population, a stratified sampling approach was incorporated to ensure representation across CVAD types and primary diagnoses, based on a similar local historical cohort^15^ (widened to include CVADs inserted in the intensive care).

Our 2015 meta-analysis established 25% of international pediatric CVADs failed prior to completion of therapy (95% CI: 20.9–29.2) at a rate of 1.97 per 1000 catheter days (95% CI: 1.7–2.2).^2^ Accordingly, our targeted sample size was 200 CVADs, to enable accurate benchmarking of complications (5% absolute precision, 90% confidence, to establish predicted 25% failure),^16^ while facilitating exploratory model development for CVAD failure risk.

### Measures and covariates

Patient (e.g., age, primary diagnosis, comorbidities), CVAD (e.g., type, gauge, tip location, lumens), and clinician (e.g., specialty, number of attempts) characteristics are descriptively reported, and were selected due to previous association with reduced CVAD performance in pediatric or adult cohorts.^15,17,18^

CVAD performance was described by a report of CVAD failure (CVAD complications that result in permanent cessation of CVAD function, prior to completion of therapy)^2^ and CVAD complications as a composite and individual report of insertion and post-insertion complications including those which are transient: infectious (CABSI,^3^ local site infection^3^) thrombotic (venous thrombosis,^19,20^ breakage,^21^ occlusion,^22^) mechanical (dislodgement,^20^ migration^20^) and severe skin complications.^23^ All complications were defined in accordance with international contemporary literature, with a full description available via online content (Supplementary Material 1).

Clinical practice variation was compared to practices with strong evidence. Specifically positive practices were ultrasound guidance for CVAD insertion^9^ (by proceduralist; documented in the medical record); tip placement not outside of the cavo-atrial junction or right atrium (assessed via imaging);^12^ and appropriate device selection^13^ (including no insertion of implanted devices during active infection [i.e., positive blood cultures],^24^ PICCs for short-term [<7 days] peripherally-compatible infusates,^13^ totally implanted devices for infants and neonates [<1 year].^13^)

### Data sources

Operating theatre lists and ICU admission records (i.e., where CVADs were inserted) were screened Monday-to-Friday by specialized research nurses to identify eligible patients. The patients were assessed bi-weekly until the CVAD was removed or for 3 months. The assessments were carried out by the research nurses either in person (while still admitted to QCH) or over the phone (while discharged or at an alternative site; a process we have previously used reliably),^25^ with additional data sourced from the patient’s electronic medical record. All data (including healthcare utilization) were collected using a dedicated, secure, web-based REDCap (Research Electronic Data CAPture, Vanderbilt) database.^26^

### Bias

Selection bias was minimized by participants being selected via an equitable inclusion criterion with sequential recruitment (based on operating theatre lists and ICU admission records), however, some participants were missed due to staff availability (i.e., weekends, after-hours) and maximum recruitment being reached, but participant sampling distribution was prospectively matched across device types and primary diagnosis with historical local CVAD databases (widened to include the intensive care setting).^15^ Information bias was decreased by having data collected by dedicated, experienced clinical research nurses (including established inter-rater reliability of data collection processes),^25^ clear and rigorous outcome definitions (including CVAD performance outcome assignment by infectious disease physicians or radiologists, when appropriate), and prospective methods (eliminating recall bias).^27^

### Healthcare cost estimation

The cost for CVAD failure, CVAD complication, infectious complications, thrombotic complications, dislodgement, insertion-related complication, and severe skin complications were estimated from a health care system perspective. The primary cost outcome is the expected cost per 1000 CVADs calculated as the product of the incidence rate for 1000 CVADs and the estimated cost per episode (CVAD failure, CVAD complication, infectious complications, thrombotic complications, dislodgement, insertion-related complication, and severe skin complications). The cost per episode includes the cost of devices (including replacement devices), dressings and securement materials, imaging guidance (X-ray or ultrasound), inpatient hospital stay, operating room, pathological tests, medications, and time of nursing and medical staff. Utilization of each resource, including self-reported estimates of time taken to treat was based on data collected within the study and entered using the REDCap database. The cost per episode was estimated based on resource utilization multiplied by unit prices (Supplementary Material 3). Prices for salary and other items were based on prices faced by local public hospitals collected as part of this study from the site hospital and from previously published estimates of pediatric hospital-specific prices for materials associated with device insertion where such information was previously known.^28,29^ The cost of CABSI and venous thromboembolism (VTE) complications were based on national average cost estimates of these complications.^30^ All costs are reported in 2020 Australian dollars (A$).

### Statistical analysis

Data collected were thoroughly cleaned and checked for accuracy (10% by second research nurse) prior to importing into Stata (version 13; StataCorp, College Station, TX) for statistical analysis. The CVAD was the unit of measurement, with some children having multiple CVADs within the cohort. Descriptive statistics for normally distributed (mean and standard deviation) and non-normally distributed (median and interquartile range [IQR]) are reported for clinical characteristics of included patients. The proportion and associated 95% confidences intervals (95% CI), cost per episode, and cost per 1000 CVADs, are reported for CVAD-associated complications and serious adverse events, and variations in care. Missing data were not imputed. Kaplan–Meier estimates were used to estimate the probabilities of CVAD failure and complication. Univariable and multivariable analyses of the association between CVAD device types and time to first CVAD failure were performed with Cox proportional hazards model with shared frailty term set at participant’s level to account for intra-subject correlation (hazard ratios (HR) reported). Covariates were selected based on relationship laid out on Directed Acyclic Graphs (DAG) using clinical knowledge and knowledge acquired from previous studies and consisted of age, previous device, previous vessel occlusion, and primary diagnosis (Supplementary Material 2).^15,17,18,31^ Clinical practice variation compared to practices with strong evidence (was explored descriptively across clinical characteristics (e.g., primary diagnosis, catheter types)).

## Results

### Participants

**Fig. 1:**
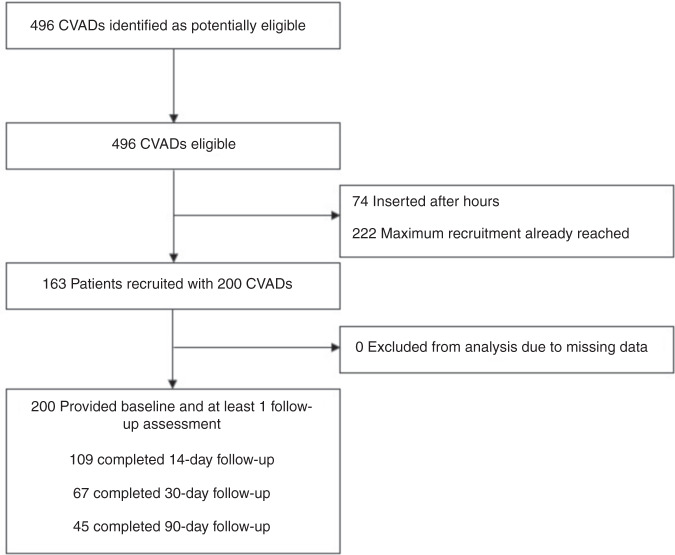
Flow diagram of study participation. CVADs Central venous access devices.

As described in Fig. 1, 496 CVADs were inserted over the study period. From this sample, 296 were missed due to research nurse availability. Finally, 163 patients with 200 CVADs were recruited and followed for 6993 catheter days.

The most commonly inserted CVADs were PICCs (*n* = 119; 60%; 2612 catheter days) (Table 1). However, totally implanted venous devices and tunneled cuffed CVAD were often in situ at 3-month study end, and thereby contributed considerable catheter days (TIVD: *n* = 21; 10%; 1659 catheter days; tunneled cuffed: *n* = 25; 13%; 1895 catheter days). Short-term CVADs (e.g., PICCs, non-tunneled CVADs) were mainly inserted for children with respiratory conditions and surgical (including cardiac) procedures, while long-term CVADs (e.g., TIVD, tunneled cuffed) were predominantly used for children with oncological, hematological, and gastroenterological conditions. Over a quarter of the cohort had received >5 previous CVADs (*n* = 28; 27%), and many had known occluded vessels limiting CVAD insertion locations. Multiple insertion attempts were necessary for 14% of insertions (*n* = 26; 14%) mainly for PICCs and tunneled cuffed CVADs, however, image guidance was not consistently used for the insertion of TIVD, tunneled cuffed and HD catheters, and catheter tip placement outside of the cavo-atrial junction was evident in 43 CVADs (22%).

**Table 1:** Participant and device characteristics at insertion (*n* = 200).

Variable	PICC	TIVD	Non-tunnel	Non-tunnel HD	Tunnel non-cuff	Tunnel HD	Tunnel cuff	Total
CVAD Catheter days	119 (60) 2680	21 (10) 1659	18 (9) 151	5 (3) 40	7 (4) 276	5 (3) 292	25 (13) 1895	200 6993
*Patient characteristics*								
Age								
Median [years] (IQR)	5 (2–10)	3 (3–10)	0.5 (0.1–5)	0.9 (0.5–5)	0.3 (0.2–3)	3 (3–16)	4 (1–8)	4 (0.1–10)
Neonates (0–30 days)	0	0	1 (6)	0	0	0	1 (4)	2 (1)
Infants (31 to <1 year)	15 (13)	1 (5)	10 (56)	3 (60)	5 (71)	0	5 (20)	39 (20)
Children (1 to <12 years)	80 (67)	18 (86)	7 (39)	1 (20)	2 (29)	3 (60)	15 (60)	126 (63)
Adolescent (12–18 years)	24 (20)	2 (10)	0	1 (20)	0	2 (40)	4 (16)	33 (17)
Primary diagnosis^a^								
Respiratory (non-CF)	52 (44)	3 (14)	0	0	1 (14)	0	0	56 (28)
Oncology and hematology	17 (14)	17 (81)	1 (6)	3 (60)	0	1 (20)	16 (64)	55 (28)
Cystic fibrosis	30 (25)	3 (14)	0	0	0	0	0	33 (17)
General surgical	12 (10)	6 (29)	4 (22)	0	0	1 (20)	5 (20)	28 (14)
Gastroenterology	8 (7)	0	4 (22)	2 (40)	2 (29)	3 (60)	6 (24)	25 (13)
Coronary care/cardiac	8 (7)	0	4 (22)	0	1 (14)	1 (20)	1 (4)	15 (8)
Hepatic	3 (3)	0	5 (28)	2 (40)	2 (29)	0	1 (4)	13 (7)
Other	16 (13)	1 (5)	4 (22)	0	0	5 (100)	5 (20)	31 (16)
Previous CVADs (no.)								
1	14 (27)	8 (53)	3 (33)	0	0	0	9 (60)	34 (32)
2–3	15 (29)	4 (27)	4 (44)	2 (50)	1 (20)	1 (20)	2 (14)	29 (27)
4–5	7 (14)	2 (13)	1 (11)	0	1 (20)	1 (20)	2 (14)	14 (14)
>5	16 (31)	1 (7)	1 (11)	2 (50)	3 (60)	3 (60)	2 (13)	28 (27)
Known occluded vessels^a^								
Basilic	15 (87)	2 (10)	2 (11)	0	3 (43)	0	3 (12)	25 (13)
Brachial	4 (4)	1 (5)	0	0	1 (14)	0	1 (4)	7 (4)
Cephalic	2 (2)	1 (5)	0	0	0	0	1 (4)	4 (2)
Axillary	2 (2)	1 (5)	1 (6)	0	2 (29)	0	2 (8)	8 (4)
Internal jugular	1 (1)	0	1 (6)	2 (40)	1 (14)	1 (20)	1 (4)	7 (4)
Other (e.g., subclavian)	4 (4)	0	2 (12)	2 (40)	1 (14)	1 (20)	1 (4)	11 (7)
*Device characteristics*								
Vessel								
Basilic	85 (71)^b^	0	0	0	0	0^b^	0	85 (43)
Brachial	18 (15)	0	0	0	0	0	0	18 (9)
Cephalic	5 (4)	0	0	0	0	0	0	5 (3)
Axillary	8 (7)	0	0	0	0	0	0	8 (4)
Internal jugular	–	16 (76)	11 (61)	2 (40)	7 (100)	4 (80)	20 (80)	60 (30)
Subclavian	–	5 (24)	2 (11)	0	0	0	5 (20)	12 (6)
Femoral	2 (2)	–	5 (28)	3 (60)	0	0	0	10 (5)
Lumen numbers								
One	103 (87)	21 (100)	1 (6)	0 (0)	2 (29)	0 (0)	4 (16)	131 (66)
Two	16 (13)	0 (0)	2 (11)	5 (100)	5 (71)	5 (100)	20 (80)	53 (27)
Three	0 (0)	0 (0)	15 (83)	0 (0)	0 (0)	0 (0)	1 (4)	16 (8)
*Clinician characteristics*								
Multiple insertion attempts								
2	14 (12)	1 (5)	0^c^	0	0	0	4 (16)^b^	19 (10)
≥3	5 (4)	0	0	0	1 (14)	0	1 (4)	7 (4)
Guidance								
Ultrasound	118 (99)	11 (52)	15 (83)^b^	4 (80)	7 (100)	2 (40)	11 (44)	166 (83)
X-ray for confirmation	114 (96)	21 (100)	8 (44)	2 (40)	7 (100)	5 (100)	22 (88)	179 (90)
X-ray during placement	5 (4)	4 (19)	0	0	0	1 (20)	8 (32)	18 (9)
Cut down	0	0	1 (6)	0	0	0	4 (16)	6 (3)
Tip position								
Cavo-atrial junction	104 (87)	5 (24)^d^	1 (6)^c^	1 (20)^c^	5 (71)	2 (40)	7 (28)^b^	125 (63)
Superior vena cava	12 (10)	8 (38)	5 (29)	0	2 (29)	1 (20)	13 (52)	41 (21)
Right atrium	0	6 (29)	5 (29)	1 (20)	0	2 (40)	4 (16)	18 (9)
Non-central	1 (1)	0	1 (6)	0	0	0	0	2 (1)
Inferior vena cava	2 (2)	0	2 (12)	0	0	0	0	4 (2)
Procedural time								
Median [min] (IQR)	62 (46–82)	77 (58–89)	162 (78–298)	40 (40–57)	92 (74–119)	129 (71–149)	80 (66–137)	70 (53–98)
Dwell								
Median [day] (IQR)	14 (11–23)	>90 (>90)	7 (1–13)	3 (1–13)	26 (9–77)	90 (8–>90)	90 (71–>90)	16 (11–63)
In situ at study end (3 months)	6 (5)	17 (81)	0	0	1 (14)	3 (60)	18 (72)	45 (23)

*CVAD* central venous access device, *HD* hemodialysis, *IQR* Interquartile range, *PICC* peripherally inserted central catheter, *TIVD* totally implanted venous device.

^a^Multiple response options.

Missing data: ^b^1 ^c^3 ^d^2.

### CVAD performance

**Fig. 2:**
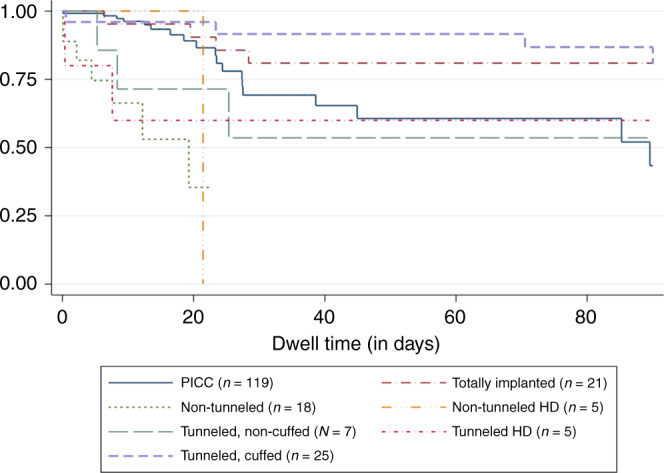
Kaplan–Meier survival estimates of CVAD failure per device type. HD Hemodialysis, PICC Peripherally inserted central catheter.

As reported in Table 2, CVAD failure was 20% (*n* = 30; 95% CI: 15–26), at an IR of 5.72 per 1000 catheter days (95% CI: 4.09–7.78). While failure proportion was highest in tunneled, non-cuffed (43%; *n* = 4), tunneled HD (40%; *n* = 2), and non-tunneled (39%; *n* = 7) CVADs, non-tunneled CVADs had the highest incidence rate per 1000 catheter days (46.35; 95% CI: 18.83–93.18). As displayed in Fig. 2, failure was common earlier in the device dwell for non-tunneled and permanent HD (i.e., <20 catheter days), while failure of other devices was frequently later (i.e., ≥20 catheter days).

**Table 2:** CVAD performance (*N* = 200; 6993 catheter days) and projected economic cost.

	Proportion of CVADs (%)	95% CI	Episodes (*N*)	Incidence rate per 1000 catheter days	95% CI	Cost per episode	95% CI	Cost per 1000 CVADs
CVAD failure	20	15–26	40	5.72	4.09–7.78	A$826	A$762–A$890	A$165,372
Tunneled, non-cuffed (*n* = 7)	43	9–82	3	10.87	2.25–31.44	A$824	A$591–A$1060	A$354,320
Tunneled HD (*n* = 5)	40	5–85	2	6.85	0.83–24.52	A$889	$581–A$1200	A$355,600
Non-tunneled (*n* = 18)	39	17–64	7	46.35	18.83–93.18	A$569	A$464–A$674	A$221,910
Non-tunneled HD (*n* = 5)	20	0–72	1	24.68	0.63–131.58	A$569	A$291–A$847	A$113,800
Totally implanted (*n* = 21)	19	5–42	4	2.41	0.66–6.16	A$1464	A$1110–A$1820	A$278,160
Tunneled, cuffed (*n* = 25)	16	5–36	4	2.11	0.58–5.40	A$824	A$622–A$1030	A$131,840
PICC (*n* = 119)	16	10–24	19	7.09	4.27–11.05	A$649	A$576–A$722	A$103,840
CVAD complication^a^	43	36–50	86	12.29	9.84–15.16	A$2327	A$2200–A$2450	A$1,000,610
Infectious complications								
CABSI	5	2–9	10	1.43	0.69–2.63	A$14,943^b^	A$12,600–A$17,300	$749,150
Local infection	0	0	0	0	0	$0	$0	$0
Thrombotic complications								
Occlusion	25	19–31	49	7.00	5.19–9.25	A$239	A$223–A$256	A$59,750
Breakage	4	2–8	8	1.14	0.49–2.25	A$381	A$315–A$447	A$15,240
Venous thrombosis	3	1–6	5	0.72	0.23–1.67	A$7045^b^	A$5510–A$8590	A$211, 350
Dislodgement								
Partial dislodgement	16	11–21	31	4.43	3.01–6.29	A$43	A$39–A$47	A$30,400
Complete dislodgement	2	1–5	4	0.57	0.16–1.46	A$1469	A$1110–A$1830	A$29,380
Insertion-related complication	1	0–3	2	0.20	0.00–1.03	A$09	A$5–$13	A$90
Severe skin complication	8	4–12	15	2.15	1.20–3.53	A$1533	A$1340–A$1730	A$122,640

^a^One device can have more than one complication type; expected cost per episode adjusted to include only one replacement device in instances of multiple complications.

^b^Source: National Hospital Cost Data Collection. Public Hospitals Cost Report, Cost Weights, for Ar-Drg Version 8.0 Round 22 Sydney: IHPA (2020).

CVAD complications were evident in 43% of CVADs (*n* = 86; 95% CI: 36–50), at a rate of 12.29 per 1000 catheter days (95% CI: 9.84–15.16). The most common complications during CVAD dwell were occlusion (25%; *n* = 49 [95% CI: 19–31]; IR 7.00 [95% CI: 5.19–9.25]) and partial dislodgement (16%; *n* = 31 [95% CI: 11–21]; IR 4.43 [95% CI: 3.01–6.29]). While insertion-related complications were uncommon, several significant skin complications (e.g., surgical wound dehiscence, allergic dermatitis) were evident (8%; *n* = 15 [95% CI: 4–12]; IR 2.15 [95% CI: 1.20–3.53])

The expected costs associated with CVAD failure were A$826 per episode, and A$ 165,372 per 1000 CVADs. The highest CVAD failure costs per episode was A$1464 for totally implanted devices, however, per 1000 CVADs costs were highest with tunneled HD (A$354,320) and non-tunneled CVADs (A$355,600). This reflects the higher incidence of failure associated with these procedures. Total CVAD complication costs per 1000 CVADs was A$1,000,610. Per episode, costs were greatest for CABSI at A$14,943, and per 1000 CVADs (A$749,150). Further significant episode level costs were associated with venous thrombosis (A$7045), complete dislodgement (A$1469), and severe skin complications (A$1533).

The results of the univariable and multivariable regression for risk of CVAD failure are available in Supplementary Material 4. Univariable cox-regression results showed a strong association (*p* < 0.05) of CVAD failure with age (HR 0.92; 95% CI: 0.86–0.99) and non-tunneled CVADs (HR 6.82; 95% CI: 2.70–17.20). However, only non-tunneled CVADs (HR 4.27; 95% CI: 1.49–12.18; referent PICC) had a statistically significant association in CVAD failure in the final multivariable analysis.

### Variations in care

Variations between recommended and actual care are described in Table 3. Ultrasound guidance for insertion was not used for 16% (*n* = 32) CVADs, mainly for tunneled, cuffed (*n* = 14; 56% of tunneled, cuffed CVADs inserted) and totally implanted devices (*n* = 10; 48% of totally implanted devices inserted). Sub-optimal tip-positioning (outside of the cavo-atrial junction or right atrium) was evident in 22% (*n* = 43) of the cohort, primarily for PICCs (*n* = 13; 11% of PICC inserted) and tunneled, cuffed (*n* = 13; 52% of tunneled, cuffed inserted). Over 10% of PICCs (10.5%; 21% of PICCs inserted) had fewer than seven days of peripherally-compatible IV therapy, for children with orthopedic (*n* = 7), neurological (*n* = 5), infectious disease (*n* = 3), ear, nose and throat (*n* = 3) and burns (*n* = 2).Only one totally implanted device was inserted for an infant receiving treatment for oncology/hematology.

**Table 3:** Variations in care (*n* = 200).

Variation	Device characteristics	*N* (%)^a^	Patient characteristics	*N* (%)^b^
No ultrasound guidance for insertion		32 (16)		
Device	Tunneled, cuffed	14 (56)	Oncology/hematology	9 (64.3)
			General surgical	4 (28.6)
			Gastroenterology	4 (28.6)
			Orthopedic	1 (7.1)
			Other	4 (28.6)
	Totally implanted	10 (48)	Oncology/hematology	9 (64.3)
			General surgical	3 (30.0)
			Cystic fibrosis	1 (10.0)
			Respiratory (non-CF)	1 (10.0)
	Non-tunneled	3 (17)		
	Permanent HD	3 (60)		
	PICC	1 (<1)		
	Temporary HD	1 (20)		
Sub-optimal tip-positioning		43 (22)		
Device	PICC	13 (30)	Other respiratory	6 (46)
			Oncology/hematology	2 (15)
			Other	2 (15)
			Gastroenterology	2 (15)
			Cystic fibrosis	1 (6)
			Hepatic	1 (6)
			General surgical	1 (6)
	Tunneled cuff	13 (30)	Oncology/hematology	10 (77)
			General surgical	3 (23)
			Gastroenterology	2 (15)
			Other	2 (15)
	Totally implanted	8 (19)		
	Non-tunneled	6 (14)		
	Tunneled, non-cuffed	2 (4)		
	Permanent HD	1 (2)		
Devices inserted with current infection		27 (14)		
Device	PICC	83 (70)		
	Non-tunneled	9 (50)		
	Totally implanted	3 (14)		
	Permanent HD	2 (40)		
	Tunneled, non-cuffed	1 (14)		
	Tunneled, cuffed	4 (16)		
	Temporary HD	0 (0)		
PICCs < 7 day therapy with peripherally-compatible infusates (*n* = 119)		21 (11)	Orthopedic	7 (33)
			Neuro	5 (24)
			Respiratory (non-CF)	3 (14)
			Other	17 (81)
Neonates and infants with totally implanted devices		1 (<1)	Oncology/hematology	1 (100)

*CF* cystic fibrosis, *HD* hemodialysis, *PICC* peripherally inserted central catheter.

^a^Denominator of proportion (%) is the device type sample.

^b^Denominator of proportion (%) is the case device type sample.

## Discussion

CVADs are a vital component of modern healthcare provision. This is the first study to comprehensively and prospectively describe CVAD insertion practices, performances, and healthcare-costs across a single large pediatric health service. From these data, we have a better understanding of the current insertion, performance, and cost burden of CVADs in pediatric healthcare. We also identified key opportunities to improve CVAD performance, both locally and internationally.

Overall, CVAD failure prior to completion of therapy in this cohort is similar to previous international estimates,^2^ however this early evaluation of new CVAD types (i.e., tunneled, non-cuffed CVADs) demonstrate challenges (*n* = 7; 43% failure; 11.1 per 1000 catheter days). This may be because these new devices are being implemented as a “rescue” device, when other, traditional CVAD routes are no longer available or additional lumens are necessary for small vasculature, and further data regarding their performance are necessary. Non-tunneled, short-term devices, such as non-tunneled CVADs (inserted in the jugular, subclavian or femoral vein) and non-tunneled HD catheters were associated with the highest rate of failure, per 1000 catheter days (49.3 per 1000 catheter days; 27.0 per 1000 catheter days; respectively). This should not be an acceptable outcome of clinical practice.

As evident in other recent descriptions,^15,31–33^ CABSI rates have reduced in recent decades, being 5% in our cohort, however other CVAD complications, especially occlusion and partial dislodgment, are high (25%, 7.2 per 1000 catheter days; 16%, 4.6 per 1000 catheter days; respectively), and should become the focal point of innovation and improvement. The prevention of occlusion needs to encompass the range of occlusion pathologies; including thrombotic (i.e., insertion technique, tip position, catheter materials), infusate precipitation (i.e., flushing, compatibility mapping) and mechanical (i.e., tip position, securement, quality external equipment) causes. Similarly, partial dislodgement innovations need to center on effective securement and tip-positioning.^31,34,35^ All causes of CVAD failure result in treatment disruption—and this has been consistently demonstrated to be associated with reduced survival for children with cancer.^36–38^

The financial costs of CVADs in healthcare have previously centered on CABSI^4,7,8^ and, less-often, thrombosis.^39^ As demonstrated in our study, the financial benefits of reducing the incidence of CABSI remain considerable. However, all aspects of CVAD failure (including the removal and insertion procedure) and complications (primarily treatment; but also need for replacement devices for some complications) are associated with immediate hefty costs. Our results are based on a combination of prospectively collected data and established estimates, including the Australian National Hospital Costing Data Collection.^40^ These are likely to be an under-estimation of costs as they fail to incorporate long-term health consequences, including increased morbidity and mortality associated with complications. However, conservatively for 1000 CVAD procedures, the CVAD complications cost is more than A$1,000,000 annually. These costs are not only associated with CABSI and venous thrombosis, and should help direct policy makers in the future. Consequently, the prevention of CVAD failure and complications is a ripe area for investment with significant potential for high returns in terms of both improved quality of treatment and reduction in healthcare resources.

Exploring gaps between current practice and best practice, highlight many opportunities for correction and invention. The sub-optimal catheter tip placement described in the study (22%; *n* = 43) was occurred more frequently than desired, and is also likely to be an underestimate of true events, as it relied on accurate interpretation of radiological images (i.e., chest X-ray, fluoroscopy). These are relatively subjective, and easily influenced by image acquisition and respiratory movement.^41,42^ Sub-optimal tip placement is a key risk factor in the development of many catheter complications; including thrombosis, non-thrombotic occlusion, arrythmia, and cardiac erosions. Technologies, such as intracavitary electrocardiography-based PICC tip confirmation, show potential to reduce insertion, and post-insertion-related complications, in addition to other procedural improvements (e.g., faster placement, reduced cost).^42,43^

PICCs have been highlighted as a device at risk of over- or inappropriate use, resulting in preventable harm.^44,45^ Within this cohort we identified PICCs as having the lowest proportion of device failure (16%; 95% CI: 10–24), but a moderate incidence rate of failure (12.7 per 1000 catheter days). Inappropriate use was evident, with 21 PICCs (10.5% of PICC cohort) inserted for a peripherally-compatible therapy administration of fewer than 7 days, potentially placing these patients at preventable and increased risk for thrombosis and infection.^13^ The indications for safe and appropriate use of PICCs and other CVADs were recently defined in the Michigan Appropriateness Guideline for Intravenous Catheters in pediatrics (miniMAGIC), and should be used as a baseline standard of care.^13^ Totally implanted devices inserted in neonates and infants are generally inappropriate, due to the risk of wound dehiscence due to lack of adipose tissue.^13,46^ The appropriate use of all vascular access devices can play a vital role in vessel health preservation, and complication prevention, for chronic, acute, and critically ill children.^13^

### Limitations

This study has limitations, primarily related to it being based in a single, metropolitan, tertiary pediatric facility, limiting generalizability to other hospitals, settings, and diagnostic groups. Also, not all children with CVADs were able to be recruited, which may have introduced sampling bias related to lack of after-hours recruitment. Additionally, we were not able to recruit neonates immediately after birth (admitted for birth-associated injuries or conditions), since these neonates are cared for at a maternity hospital adjacent to the pediatric hospital. However, the prospective design over multiple data points (including 6787 catheter days), overall transparency, and rigorous, and internationally benchmarked outcome measures strengthen the study’s reliability and validity. Lastly, the cost estimates were potentially skewed (i.e., low frequency and very high cost) in some cases given the lack of sufficient sample sizes.

## Conclusion

This study has provided a comprehensive explanation of CVAD contemporary practice, performance, and value in a tertiary-referral pediatric hospital. We have highlighted several tangible practice areas that should be targeted towards improved clinical and health services outcomes, which are likely to have relevance to many pediatric hospitals. In particular, we recommend further research and clinical practice improvement surrounding the integration of new CVAD types (e.g., tunneled, non-cuffed CVADs), CVAD pathology-based occlusion and dislodgment strategies, the appropriate use of PICCs, and the potential for tip-positioning technologies. Further investment in these key diverse practices will likely lead to extensive benefit for health services, both financially and clinically.

## Supplementary information

Supplementary Materials
