# Supplementary material for: Pediatric central venous access devices: practice, performance, and costs
Source: Pediatr Res. 2022 Feb 8;92(5):1381–90. doi: 10.1038/s41390-022-01977-1 (PMC9700519; doi:10.1038/s41390-022-01977-1)
Supplement: Supplementary file 1 — Supplementary Materials [file 41390_2022_1977_MOESM1_ESM.pdf]

Supplementary Material 1: Definitions of CVAD complications

Variable	Definition
**Catheter-associated bloodstream infection (CABSI)**	A laboratory confirmed bloodstream infection (BSI) where an eligible BSI organism is identified with a CVAD in place for >2 consecutive calendar days on the day of the BSI (day of CVAD placement being Day 1) and the CVAD was in place on the date of the event or the day before, (see CDC Device-associated Module BSI for full criteria)[3] confirmed by an infectious disease specialist.
**Local infection**	Clinical signs of local venous infection at the CVAD insertion site (e.g., purulent drainage), confirmed with a positive (>15cfu) swab or catheter tip, but with negative or no blood culture (see CDC VASC definition for full criteria for full criteria)[3] confirmed by an infectious disease specialist.
**Venous thrombosis**	Ultrasound/venographic/image confirmed thrombosed vessel at CVAD location, in a symptomatic patient (pain, swelling.[17, 18]
**Breakage**	Visible split in CVAD material, evidence of external fluid leakage or radiographic evidence of extravasation/infiltration into tissue [19]
**Occlusion**	Unable to inject or aspirate from the CVAD[20]
**Dislodgement and migration**	*Partial:* Change in CVAD length at insertion site (inner catheter visible), displacement of the CVAD tip so it is no longer central, however remains in the vein, or displacement of the TIVD needle from the reservoir. *Total*: CVAD completely leaves the vein or TIVD reservoir displacement.[18]
**Severe skin complication**	Allergic dermatitis, extravasation or surgical wound dehiscence

BSI: Bloodstream infection; CDC: Centers for Disease Control and Prevention; CFU: Colony forming units; CVAD: Central venous access device; TIVD: Totally implanted venous device; VASC: Vascular access site complication

Supplementary Material 2: Directed Acyclic Graphs for variable selection.

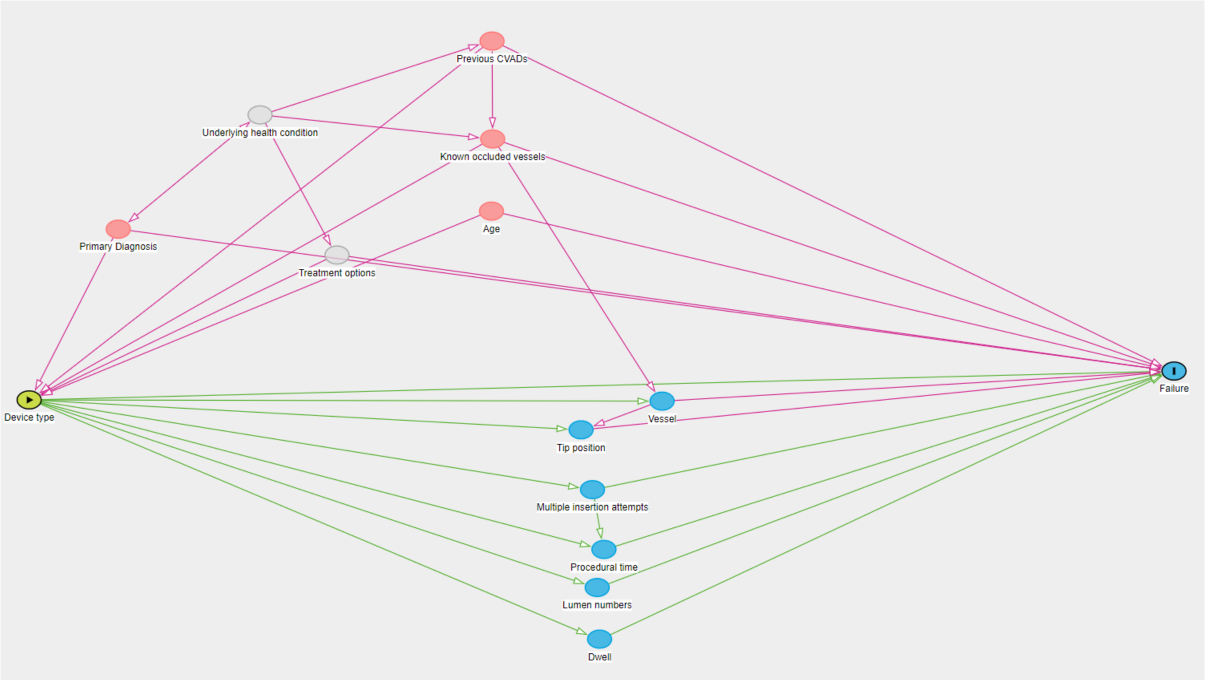

CVAD: Central venous access device

Supplementary Material 4: Unit costs used in the study (2020 A$)

**Resource item**	**Unit**	**Unit cost**
**Staff**		
Registered nurse	Per minute	$0.85
Registered Medical Officer	Per minute	$1.21
**Service**		
Ultrasound	Per item[a]	$117.20
X-ray	Per item	$40.10
Blood culture	Per item[b]	$4.55
**Equipment**		
PICC	Per item	$140
Tunnelled catheter	Per item	$315
Tunneled HD (Hickman Catheter®)	Per item	$185
Non-tunneled catheter	Per item	$60
Totally implanted	Per item	$450
Gloves – non sterile	Per item	$1.22
Gloves – sterile	Per item	$0.05
Saline ampule	Per item	$0.05
10ml syringe	Per item	$0.09
1ml syringe	Per item	$0.24
3-way tap (no extension)	Per item	$0.62
Alcohol/ CHG swab (small square)	Per item	$0.12
Alcohol/ CHG swab (small stick)	Per item	$0.24
Prefilled syringe	Per item	$0.42
Needleless connector	Per item	$1.35
Clamp	Per item	$2.87
Dressing pack	Per item	$0.46
Drawing up needle	Per item	$0.02
Bottle CHG – 30ml	Per item	$1.93
Sterile gauze	Per item	$0.12
Sodium Chloride 1L	Per item	$0.99
Burette	Per item	$5.54
Administration set	Per item	$8.26
Sterile scissors	Per item	$2.65
Needle	Per item	$0.13
**Medications**		
Enoxaparin sodium	Per dose	$135.44
Lignocaine and prilocaine mixture cream	Per 5g tube	$6.70
Vancomycin	Per 500 mg vial	$8.22
Alteplase	Per 2mg vial	$99.00
Ceftriaxone	Per 1g vial	$3.10
Gentamicin	Per dose	$11.17 for 80 mg/2 mL injection

[a] MBS item 13815, [b]MBS item 73802.

Available at http://www.mbsonline.gov.au/internet/mbsonline/publishing.nsf/Content/Home

CHG: Chlorhexidine gluconate, MBS: Medicare Benefits Schedule

Supplementary Material 4: Univariable and multivariable analyses of the association between CVAD device types and time to first CVAD failure (Device N= 200; Participant N=163)

	No fail	Fail	Univariable	Multivariable
			Hazard ratios (95% CI)	
**Device type** (Ref: PICC)	100 (84.0)	19 (16.0)	1.0	1.0
Totally implanted	17 (81.0)	4 (19.0)	0.40 (0.13-1.23)	0.33 (0.10-1.13)
Non-tunneled	11 (61.1)	7 (38.9)	6.82 (2.70-17.20)*	4.27 (1.49-12.18)*
Tunneled	25 (78.1)	7 (21.9)	0.53 (0.22-1.31)	0.40 (0.15-1.03)
Hemodialysis	7 (70.0)	3 (30.0)	1.50 (0.43-5.19)	1.23 (0.31-4.84)
**Age** (1-year increase): Median (IQR)	5 (8.3)	3 (4.2)	0.92 (0.86-0.99)*	0.93 (0.86-1.02)
**Previous device (No)**	80 (84.2)	15 (15.8)	1.0	1.0
Yes	80 (76.2)	25 (23.8)	1.08 (0.56-2.10)	1.35 (0.61-2.99)
**Previous vessel occlusion (No)**	129 (80.6)	31 (19.4)	1.0	1.0
Yes	31 (77.5)	9 (22.5)	0.96 (0.44-2.10)	0.89 (0.38-2.12)
**Primary Diagnosis** (Ref: Neuro, Renal, Orthopedics, and Other)	43 (82.7)	9 (17.3)	1.0	1.0
Cardiac	9 (81.8)	2 (18.2)	0.96 (0.21-4.45)	0.68 (0.14-3.30)
GI/ Liver	15 (57.7)	11 (42.3)	1.90 (0.78-4.60)	1.28 (0.47-3.48)
Other respiratory	21 (91.3)	2 (8.7)	0.65 (0.14-3.05)	0.81 (0.16-4.01)
Cystic fibrosis	31 (93.9)	2 (6.1)	0.38 (0.08-1.79)	0.62 (0.12-3.30)
Oncology/Hematology	41 (74.6)	14 (25.5)	0.64 (0.27-1.50)	1.09 (0.42-2.83)

*Statistically significant at p <0.05

Cox proportional hazards model with shared frailty term set at participant's level was used to account for intra-subject correlation.

CI: Confidence interval; IQR: Interquartile range; PICC: Peripherally inserted central catheter; Ref: Referent
